# Laparoscopic versus open emergency colonic resection for obstruction: a multicentre retrospective cohort study

**DOI:** 10.1186/s12893-026-04220-4

**Published:** 2026-09-18

**Authors:** Joline de Groof, Silvia Marchesi, Ellie Pearce, Timothy Rockall, Tomas Vedin, Erik Agger

**Affiliations:** 1 Department of Colorectal Surgery, Royal Surrey County Hospital, Guildford, UK; 2 Faculty of Medicine, Lund University, Lund, Sweden; 3 Department of Intensive and Perioperative care, Skåne University Hospital, Malmö, Sweden; 4 Minimal Access Therapy Training Unit (MATTU), Guildford, UK; 5 Department of Surgery, Skåne University Hospital, Malmö, Sweden

**Keywords:** Emergency, Colorectal surgery, Laparoscopic surgery, Bowel obstruction

## Abstract

**Background:**

Despite its established benefits in elective colorectal surgery, the adoption of laparoscopy in the emergency setting remains limited. This retrospective international multicentre study assesses laparoscopic and open colonic resection in patients with acute benign or malignant colonic obstruction.

**Methods:**

Patients were included between 2022 and 2023. Intraperitoneal free air on imaging was an exclusion criterion. Primary outcomes were the Comprehensive Complication Index (CCI) and length of stay (LOS). Secondary outcomes included postoperative pain management, re-operation, 30- and 90-day mortality, R0-resection and lymph node yield when applicable, intraoperative bleeding, anastomosis and operative time.

**Results:**

Out of 144 patients, 73 were eligible. Forty-three (58.9%) patients underwent open and 30 (41.1%) laparoscopic resections. Obstructions were equally distributed between the right and left colon. Patients in the open group were older (77.1 vs. 61.7 years, *p* < 0.001). Clavien-Dindo II-V complications were more common (69.8% vs. 33.3%, *p* = 0.002) and the CCI was higher (27.1 vs. 7.9, *p* < 0.001) after open surgery. LOS was significantly longer after open surgery (14 (IQR 8–19) vs. 6 (IQR 5–9) days (*p* < 0.001)). Postoperative pain was managed with patient-controlled opioids and/or oral analgesics in 86.7% after laparoscopic surgery while an epidural was used in 76.7% after open surgery (*p* < 0.001). Other secondary outcomes were comparable between the groups.

**Conclusion:**

Laparoscopic emergency resection in patients with acute benign or malignant colonic obstruction was associated with lower postoperative morbidity, shorter LOS and less intensive postoperative analgesic requirements in a selected cohort. However, these findings should be interpreted cautiously given substantial differences in baseline characteristics and surgeon specialization between groups.

**Supplementary Information:**

The online version contains supplementary material available at https://doi.org/10.1186/s12893-026-04220-4.

## What does this paper add to the literature?

This multicentre study suggests that laparoscopic emergency resection can be performed safely in selected patients with colonic obstruction. The observed associations with improved postoperative outcomes, including reduced complications and shorter hospital stay may partly reflect patient selection and specialist colorectal surgeon involvement.

## Introduction

In elective colorectal surgery, laparoscopy became the approach of choice in both benign and malignant colorectal disease, since several large randomized studies showed many benefits such as reduced pain, less postoperative morbidity, shorter length of postoperative hospital stay (LOS), less cost and a better quality of life [1–6]. 

A negative resection margin (R0) is an important prognostic factor for local recurrence and disease-free survival and key quality indicator in colorectal cancer surgery. Achieving an R0 resection may be more challenging in patients presenting with colonic obstruction.

Emergency surgery is generally associated with increased morbidity and mortality when compared to elective surgery, partly since there is often not much or no time to optimize or prehabilitate patients [7, 8]. It is conceivable that the reduction of surgical trauma and preservation of abdominal wall integrity that laparoscopy confers is beneficial in this high-risk patient group.

The aim of the present study was to assess postoperative outcomes following laparoscopic and open colonic resection in patients with acute benign or malignant colonic obstruction.

## Methods

### Study population

This was an international multicentre retrospective cohort study. Patients were included from three centres (Skåne University Hospital in Lund and Malmö, Sweden and the Royal Surrey NHS Foundation Trust in Guildford, UK). Patients underwent emergency surgery between 01-01-2022 and 31-12-2023. This study is reported in accordance with the Strengthening the Reporting of Observational Studies in Epidemiology (STROBE) statement (Supplement 1).

### In- and exclusion criteria

Adult patients were included when requiring emergency surgery with colonic resection for acute colonic obstruction due to benign or malignant disease. Acute colonic obstruction was defined as the presence of clinical symptoms consistent with bowel obstruction (including abdominal distension, abdominal pain, nausea/vomiting, and/or failure to pass stool or flatus) together with radiological evidence of colonic dilation. Emergency surgery was defined as an unplanned surgical procedure performed during the index hospital admission following an acute presentation.

In case of obstruction due to colorectal cancer, patients with all TNM-stages were included. Patients treated with a stent or primary stoma without colonic resection were excluded. Furthermore, patients with disseminated free air on imaging due to a non-covered perforation, a volvulus or therapy refractory IBD needing emergency resection were excluded as well. Also, patients undergoing emergency surgery due to a complication of primary surgery were excluded. Patients were grouped for analysis based on the completed surgical approach.

The participating centres followed broadly similar perioperative colorectal surgery pathways; however, no formal standardized protocol for postoperative care, analgesia, or discharge criteria was implemented across all sites. Surgeon specialty was categorized as either emergency surgeon or colorectal surgeon. Emergency surgeons were defined as general surgeons with a subspecialty other than colorectal surgery, whereas colorectal surgeons were defined as consultant or attending surgeons with formal colorectal subspecialty training and practice.

During the inclusion period, there were no predefined institutional criteria governing the choice of operative approach. The decision to perform laparoscopic or open surgery was made at the discretion of the consultant surgeon responsible for the patient’s care, based on clinical presentation, patient factors, surgeon experience, and intraoperative findings.

### Data collection

Data was manually extracted using a predefined pro forma document. Data on age at time of surgery, body mass index (BMI), American Society of Anaesthesiologists (ASA) classification and surgical specifics such as intraoperative bleeding, anastomosis and/or defunctioning stoma creation and operative time were obtained from the medical records. In case of malignant disease, the R0-resection rate and lymph node (LN) yield were assessed. Follow-up data on postoperative complications were collected during the index hospital admission, while mortality data were collected at 30 and 90 days after surgery. All variable definitions and coding procedures were established prior to data collection to ensure consistency. Missing data were infrequent and are reported for individual variables in the corresponding tables. Missing values were handled by complete-case analysis without imputation.

### Outcomes

The primary outcomes of the study were the Comprehensive Complication Index (CCI) and LOS following surgery. Postoperative complications were recorded and graded according to the Clavien-Dindo classification [9]. CCI was subsequently calculated based on all recorded postoperative complications as described and validated by Slankamenac et al. [10].

Secondary outcomes included postoperative pain management, re-operation, 30- and 90-day mortality, R0-resection and LN yield when applicable, intraoperative bleeding, anastomosis and operative time.

R0-resection was defined as clear circumferential and longitudinal surgical resection margins free from tumour infiltration during histopathological examination and macroscopically radical local resection reported by the surgeon.

### Ethics

Ethical approval was granted by the ethics authority in Sweden and did not mandate patient consent since the present study was not an interventional study and only retrospective data was analysed. For the UK cohort, the study was classified as a service evaluation by the Royal Surrey NHS Foundation Trust and therefore did not require formal research ethics committee approval under UK regulations. Patient data was anonymized. Patients were diagnosed and treated according to national guidelines and agreements.

### Statistical analysis

Data are presented as median and interquartile range (IQR) in nonparametric data and as mean and standard deviation (SD) in case of parametric data. Categorical data was presented as number of patients and proportions. The Mann-Whitney U and Student’s t-test, the chi-square test and Fisher’s exact test were used as appropriate to compare outcomes of numeric and categorical data to determine statistical significance. The Kaplan-Meier method with log-rank and Cox regression analysis was used to estimate differences in LOS in univariable and multivariable analysis. Binary logistic regression was used to assess differences in the odds of Clavien-Dindo grade II-V complications between the open and laparoscopic groups. The statistical analysis plan was developed before data extraction, however, given the limited sample size, multivariable models were restricted to a small number of prespecified clinically relevant confounders (age, sex and ASA classification) to reduce the risk of model overfitting. No formal sample size calculation was performed, as this was a retrospective cohort study including all eligible patients during the study period. A hazard ratio (HR) > 1 indicated a higher probability of discharge in the LOS analysis. A two-tailed p-value of less than 0.05 was considered significant. SPSS for Windows (version 29.0.2.0, IBM Corp., Armonk, N.Y., USA) was used for data collection and statistical analysis.

## Results

### Patient inclusion and characteristics

**Fig. 1:**
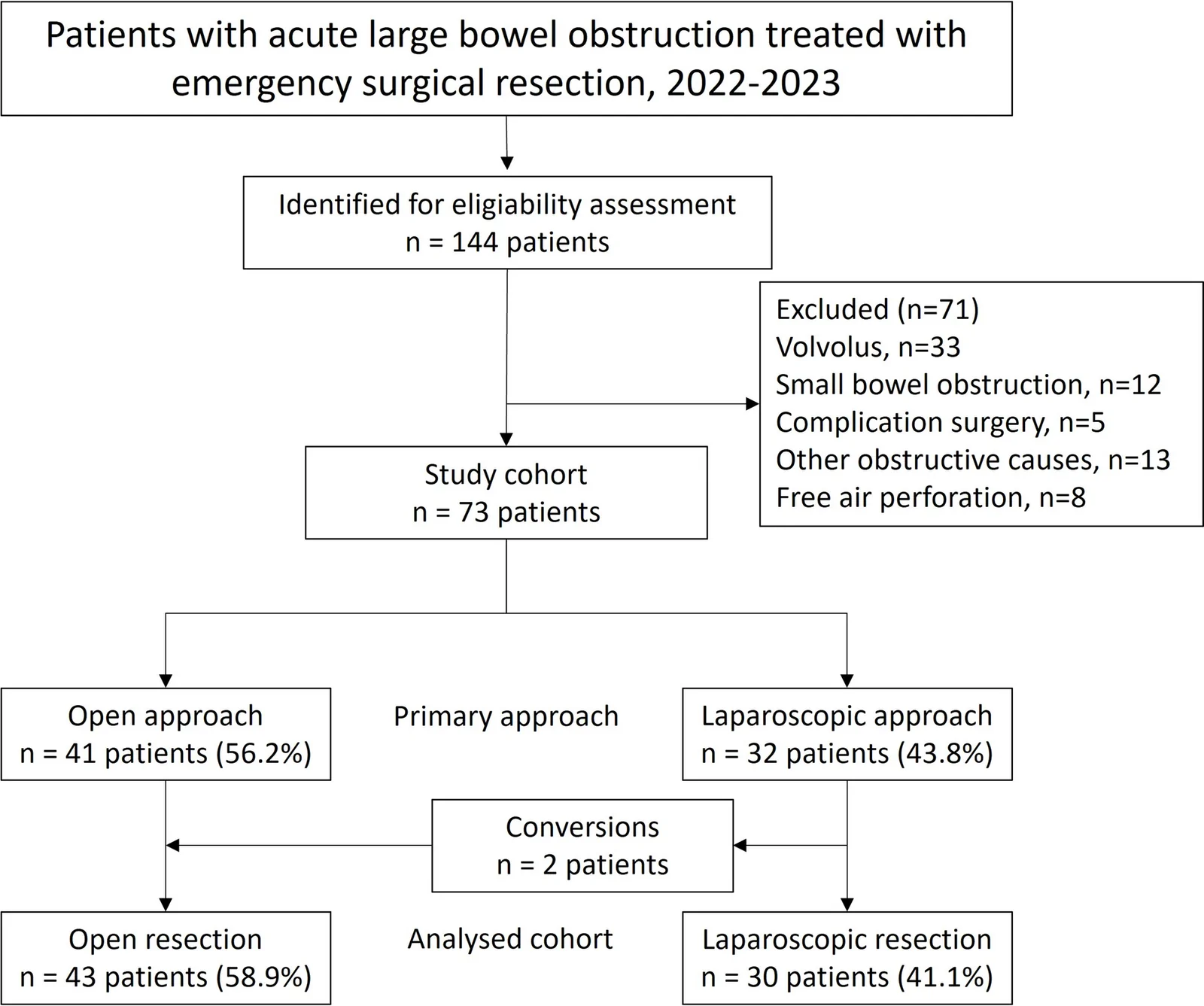
Flow diagram of patient inclusion and exclusion

In total, 144 patients were assessed and eventually 73 patients were eligible for inclusion (flowchart for inclusion in Fig. 1). Patient characteristics are presented in Table 1. Patients in the open surgery group were significantly older compared to the patients in the laparoscopic group on average. Age ranged between 40 and 95 years in the open group and between 37 and 95 years in the laparoscopic group. The majority of patients presented with an obstruction due to malignancy (69.9%) or diverticular disease (26.0%). Obstructions were equally distributed between the right and left side of the colon. In total, there were two patients, one in each group, who underwent a multivisceral resection. One patient in the open surgery group had a right hemicolectomy combined with a sigmoid resection and in the laparoscopic surgery group one patient underwent a sigmoid resection combined with salpingo-oophorectomy. There were two conversions to open surgery representing a conversion rate of 6.3% (*n* = 2/32), these patients were analysed together with the open surgery group.

**Table 1:** Patient characteristics of included patients

		All patients	Open surgery	Laparoscopic surgery	*p*-value
Number (*N*, %)		73 (100)	43 (58.9)	30 (41.1)	
Gender (N, %)	Male	25 (34.2)	14 (32.6)	11 (36.7)	0.716
	Female	48 (65.8)	29 (67.4)	19 (63.3)	
Age (mean, SD)	Years	70.5 (15.6)	77.1 (12.1)	61.7 (15.7)	< 0.001
ASA	1	6 (8.2)	1 (2.3)	5 (16.7)	0.083
	2	22 (30.1)	11 (25.6)	11 (36.7)	
	3	39 (53.4)	28 (65.1)	11 (36.7)	
	4	4 (5.5)	2 (4.7)	2 (6.7)	
	Missing	2 (2.7)	1 (2.3)	1 (3.3)	
BMI (mean, SD)		25.7 (5.3)	25.4 (5.5)	26.3 (4.9)	0.528
	Missing	8	2	6	
Obstruction	Tumour	50 (68.5)	30 (69.8)	20 (66.7)	0.588
	Diverticular disease	19 (26.0)	11 (25.6)	8 (26.7)	
	Intussusception	1 (1.4)	0	1 (3.3)	
	Ischemic stricture	3 (4.1)	2 (4.7)	1 (3.3)	
Stricture level	Right colon	41 (56.2)	25 (58.1)	16 (53.3)	0.684
	Left colon	32 (43.8)	18 (41.9)	14 (46.7)	
Caecal width	cm (mean, SD)	7.2 (2.3)	7.3 (2.3)	7.1 (2.3)	0.681
Type of resection	Right hemicolectomy	38 (52.1)	25 (58.1)	13 (43.3)	0.178
	Transverse colon resection	1 (1.4)	0	1 (3.3)	
	Left hemicolectomy	6 (8.2)	3 (7.0)	3 (10.0)	
	Sigmoid resection	26 (35.6)	15 (34.9)	11 (36.7)	
	Colectomy*	2 (2.7)	0	2 (6.7)	
Surgeon	Emergency	21 (28.8)	21 (48.8)	0	< 0.001
	Colorectal**	52 (71.2)	22 (51.2)	30 (100)	
Time of surgery	Daytime	42 (57.5)	22 (51.2)	20 (66.7)	0.187
	On call hours	31 (42.5)	21 (48.8)	10 (33.3)	
Centre	SUS Malmö	36 (49.3)	22 (51.2)	14 (46.7)	0.009
	SUS Lund	9 (12.3)	9 (20.9)	0	
	RSCH	28 (38.4)	12 (27.9)	16 (53.3)	
pT-stage	pT3a-c	23 (46.0)	13 (43.3)	10 (50.0)	0.864
	pT4a-b	27 (54.0)	17 (56.7)	10 (50.0)	
	No malignancy (n)	23	13	10	
Tumour stage	2	11 (22.0)	5 (16.7)	6 (30.0)	0.687
	3	24 (48.0)	16 (53.3)	8 (40.0)	
	4	15 (30.0)	9 (30.0)	6 (30.0)	
	No malignancy (n)	23	13	10	

*SD* standard deviation

*Subtotal or total colectomy procedures that could not be classified as segmental colonic resections

**Colorectal surgeon; defined as a consultant/attending surgeon with formal colorectal subspecialty practice

### Primary outcomes on complications and length of postoperative hospital stay

Clavien-Dindo II-V complications were more common after open surgery (69.8% versus 33.3%) and the mean CCI was 27.1 versus 7.9 (*p* < 0.001) after open and laparoscopic surgery, respectively (Table 2). In univariable binary logistic regression, open surgery was associated with higher odds of postoperative complications compared with laparoscopic surgery (OR 4.62, 95% CI 1.70-12.54; *p* = 0.003). After adjustment for age, sex, and ASA classification, the effect estimate was attenuated and no longer statistically significant (adjusted OR 3.21, 95% CI 0.97–10.59; *p* = 0.056). Sensitivity analysis based on the intended operative approach (intention-to-treat), yielded similar results with odds remaining higher following open surgery in univariable analysis (OR 4.61, 95% CI 1.71–12.45; *p* = 0.003) and after multivariable adjustment (adjusted OR 3.79, 95% CI 1.10-13.01; *p* = 0.034). Specifics on complications per group are listed in Table 3.

**Table 2:** Surgical and postoperative outcomes

		All patients	Open surgery	Laparoscopic surgery	*p*-value
Number		73 (100)	43 (58.9)	30 (41.1)	
Complication	Yes	40 (54.8)	30 (69.8)	10 (33.3)	0.002
any CD II-V	No	33 (45.2)	13 (30.2)	20 (66.7)	
CCI	Value (mean, SD)	19.2 (25.1)	27.1 (28.6)	7.9 (12.3)	< 0.001
	Value (median, IQR)	20.9 (0-29.6)	20.9 (0-36.2)	0 (0-20.9)	< 0.001
Discharge	POD (median, IQR)	9 (6–18)	14 (IQR 8–19)	6 (IQR 5–9)	< 0.001
Primary	PCEA	36 (49.3)	33 (76.7)	3 (10.0)	< 0.001
postoperative	PCA	12 (16.4)	7 (16.3)	5 (16.7)	
pain therapy	Intravenous and Oral	22 (30.1)	1 (2.3)	21 (70.0)	
	Rectus sheath catheter	1 (1.4)	1 (2.3)	0	
	Missing	2 (2.7)	1 (2.3)	1 (3.3)	
Reoperation	Yes	4 (5.5)	3 (7.0)	1 (3.3)	0.501
	No	69 (94.5)	40 (93.0)	29 (96.7)	
Mortality	< 30 days	5 (6.8)	5 (11.6)	0	0.053
Mortality	< 90 days	8 (11.0)	7 (16.3)	1 (3.3)	0.081
R0-resection	Yes	46 (92.0)	26 (86.7)	20 (100)	0.228
	No	4 (8.0)	4 (13.3)	0	
	No malignancy (n)	23	13	10	
LN yield	n (median, IQR)	22 (17–32)	20 (14–30)	26 (20–41)	0.032
Bleeding	ml (mean, SD)	124 (156)	154 (174)	82 (117)	0.053
	Missing (n)	3	2	1	
OP-time	Minutes (mean, SD)	196 (65)	202 (71)	188 (57)	0.390
	Missing (n)	1	0	1	
Anastomosis	Yes	42 (57.5)	23 (53.5)	19 (63.3)	0.402

*SD* standard deviation, *IQR* interquartile rangem, *PCEA* patient controlled epidural analgesia, *PCA* patient controlled intravenous analgesia, *LN* lymph node

**Table 3:** Specifics on complications. The numbers represent individual recorded complication events according to the Clavien-Dindo (CD) classification

Specifics on complications				
		All patients	Open surgery	Laparoscopic surgery
Number of recorded complications		70	60	10
**Surgical (total)**		**29**	**23**	**6**
Abdominal abscess	CD 2	2	1	1
	CD 3a	2	2	
Anastomotic leak	CD 2	2	1	1
Postoperative ileus*	CD 2	16	13	3
High stoma output	CD 2	1	1	
	CD 4a	1	1	
Postoperative abdominal bleeding	CD 3b	1	1	
Wound dehiscence	CD 2	1	1	
	CD 3b	2	2	
Rectal bleeding	CD 2	1		1
**Medical (total)**		**11**	**11**	**0**
Acute kidney failure	CD 2	3	3	
Atrial fibrillation	CD 2	3	3	
Cardiac arrest	CD 5	1	1	
Pulmonary embolism	CD 2	2	2	
Pulmonary oedema	CD 2	1	1	
	CD 3a	1	1	
**Neurological (total)**		**8**	**7**	**1**
Postoperative delirium**	CD 2	8	7	1
**Infectious (total)**		**15**	**12**	**3**
Pneumonia	CD 2	3	3	
Sepsis	CD 2	3	3	
Wound infection	CD 2	7	6	1
	CD 3a	1		1
Urinary tract infection	CD 2	1		1
**Other (total)**		**7**	**7**	**0**
Hematuria	CD 2	1	1	
Morphine intoxication	CD 2	1	1	
Upper GI-bleeding	CD 3a	2	2	
Multiorgan failure	CD 5	3	3	

* Defined as re-insertion of nasogastric tube and parenteral nutrition treatment

** Postoperative delirium/confusion in need of medication

**Fig. 2:**
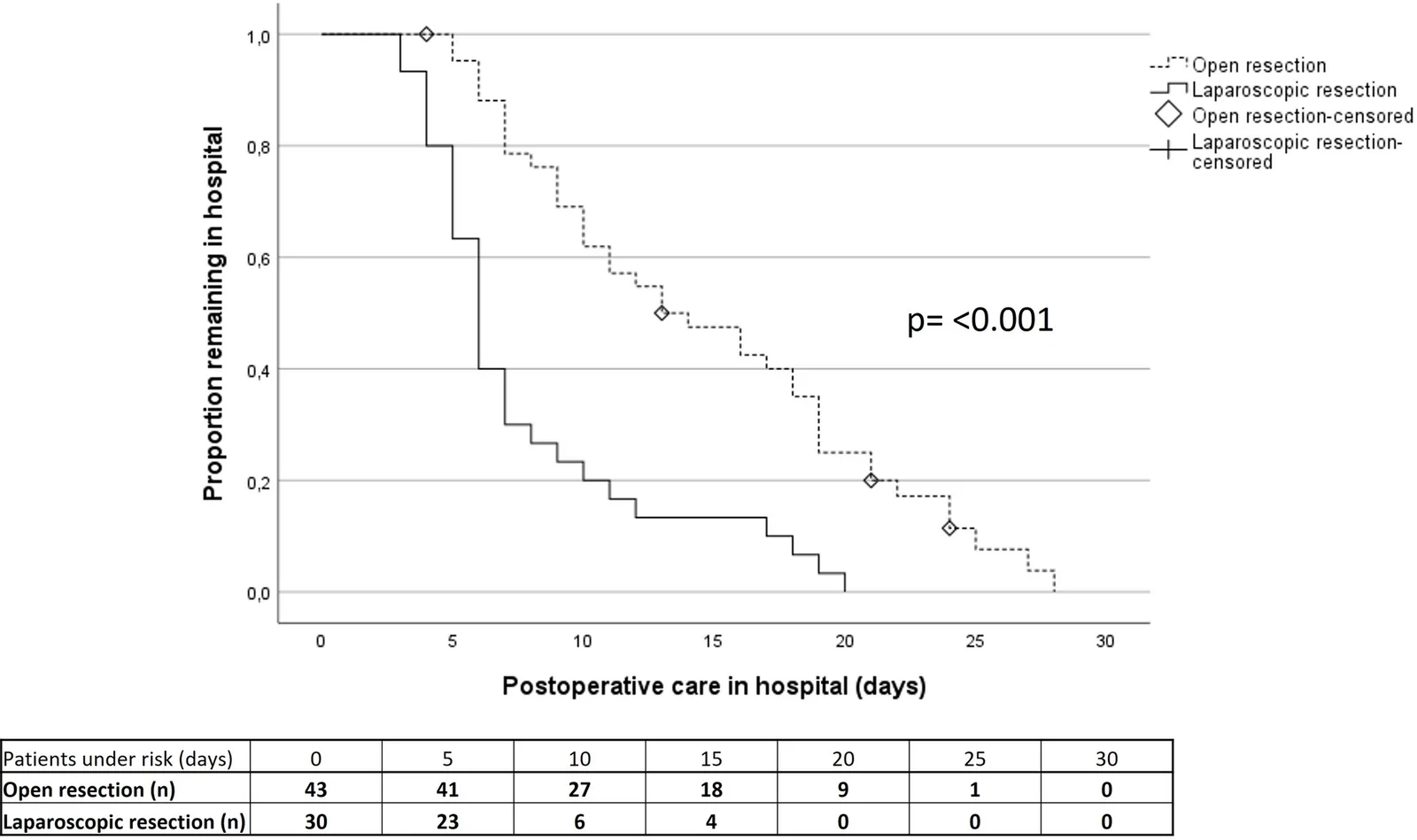
Kaplan–meier curve for postoperative length of stay

Postoperative LOS was significantly longer after open surgery at 14 (IQR 8–19) days compared to laparoscopic surgery at 6 (IQR 5–9) days (*p* < 0.001), Fig. 2. Laparoscopic surgery was associated with a significantly higher probability of discharge in both univariable (HR 1.76 (95% CI, 1.36–2.28; *p* < 0.001) and multivariable analysis (HR 1.37 (95% CI, 1.02–1.86; *p* = 0.039) adjusted for gender, age and ASA classification (Supplement 2). To assess the robustness of the primary LOS analysis, a sensitivity analysis was performed according to the intended operative approach (intention-to-treat). In univariable Cox regression, intended laparoscopic surgery was associated with a higher probability of discharge compared with open surgery (HR 1.53, 95% CI 1.20–1.95; *p* < 0.001). After adjustment for age, sex, and ASA classification, the association was attenuated and no longer statistically significant (adjusted HR 1.28, 95% CI 0.93–1.77; *p* = 0.125).

### Secondary outcomes

Secondary outcomes were comparable between the two groups. Postoperative pain was managed with patient-controlled opioid administration and/or oral analgesics in 86.7% of patients after laparoscopic surgery while epidural catheter was used in 76.7% of patients after open surgery (*p* < 0.001). Thirty-day mortality occurred in five patients in the open surgery group (11.6%) versus zero in the laparoscopic group (*p* = 0.053). A R0-resection was obtained in all patients with an obstruction due to malignancy undergoing laparoscopic surgery versus 86.7% of the patients in the open surgery group (*p* = 0.228). There were subsequently four patients with positive resection margins in the open group out of which none had a multivisceral resection. Three out of these patients had positive circumferential resection margins during histopathological examination (R1) and one patient had a macroscopically non-radical resection (R2) reported by the surgeon.

Thirty-one out of 73 (42.5%) patients were operated on during on call hours. Overall, the majority of patients (71.2%) were operated on by specialized colorectal surgeons. In the laparoscopic surgery group, all patients were operated on by colorectal surgeons. Operative time did not differ significantly between the groups. Details on secondary outcomes are presented in Table 2.

## Discussion

The present study assessed postoperative outcomes after laparoscopic and open emergency colonic resection in patients with colonic obstruction. As presented in Table 1, important baseline differences in age (77.1 years vs. 61.7 years), ASA-classification, surgeon specialization and on-call interventions existed between groups which could have affected patient selection and the observed outcomes. The low number of conversions could indicate a high degree of patient selection in the laparoscopic cohort. Additionally, the association between operative approach and time to discharge became non-significant in the adjusted intention-to-treat sensitivity analysis. Although limited by sample size, this finding suggests that differences in patient characteristics rather than operative access alone may explain part of the observed reduction in LOS after laparoscopic surgery.

Laparoscopic resection was associated with fewer postoperative complications, with a higher CCI and higher incidence of Clavien–Dindo grade II–V complications observed after open surgery. These findings are consistent with previous reports showing lower morbidity rates following laparoscopic surgery in obstructed patients [11]. In the present study, open surgery was associated with higher odds of Clavien-Dindo grade II–V complications. Following adjustment for age, sex, and ASA classification, the effect estimate was attenuated and no longer reached statistical significance. These adjusted analyses should be considered exploratory rather than causal estimates, as important confounders, including surgeon specialization and factors influencing treatment allocation, could not be fully accounted for.

Conflicting evidence exists concerning the possible effect of laparoscopy on complications; for example, one systematic review included only five studies showing fewer complications after laparoscopic surgery, whereas single-centre studies have reported no significant difference in complication rates [12]. There was no statistically significant difference in 30- and 90-day mortality in the present study, although the absolute differences observed between groups were clinically noteworthy. These mortality events may have contributed to the higher complication burden and longer postoperative hospital stay observed in the open surgery group. However, the study was not powered to detect mortality differences, and the substantial differences in patient characteristics between groups preclude causal inference regarding the effect of surgical approach on survival and a larger sample size would be needed to investigate this properly.

Several studies have shown reduced mortality in the laparoscopic setting. A previous published systematic review and meta-analysis focusing on laparoscopic and open emergency surgery for patients with colorectal disease did show lower postoperative mortality rates after emergency laparoscopic surgery [13]. In this study both patients with obstructive and non-obstructive pathology were included. These findings were mainly due to the inclusion of several large population-based studies and more recently published studies of superior quality [11, 14–17]. 

The present study found that LOS was significantly shorter following laparoscopic surgery compared to open surgery and these patients required less opiate analgesia compared to open surgery. Minimally invasive surgery is known to result in less postoperative pain and faster recovery after surgery. However, in patients with bowel obstruction, the oral delivery of analgesics may be of concern during the immediate postoperative period due to subsequent bowel paralysis. In the current study, after laparoscopic surgery as in an elective setting, the majority of patients’ postoperative pain was successfully managed without epidural analgesia.

Most patients in the open surgery group received epidural analgesia, while patients in the laparoscopic group were treated mainly with oral opioid administration supplemented with occasional intravenous doses which likely reflect perioperative treatment strategies associated with the planned surgical approach rather than objective differences in pain severity. In the laparoscopic group, no patient required escalation to epidural analgesia during recovery. However, as pain scores were not recorded and analgesic strategies were not standardized, no conclusions regarding postoperative pain severity can be drawn.

Since this was a retrospective study, the choice of operative approach was determined by the treating surgeon rather than predefined institutional criteria. Consequently, treatment allocation was likely influenced by factors that could not be fully captured in this retrospective dataset including disease severity, perceived technical complexity, surgeon preference and therefore represents an important source of unmeasured confounding. Furthermore, because all laparoscopic operations in the present study were performed by colorectal surgeons, whereas a substantial proportion of open procedures were performed by emergency surgeons, the effects of operative approach and surgeon expertise cannot be separated within this dataset. Consequently, the contribution of each factor to the observed outcomes remains uncertain. Patients in septic shock are probably more likely to undergo open surgery due to the preference of the surgeon. This is in accordance with the recommendations of the World Society of Emergency Surgery, where an open approach is advised in patients presenting with septic shock [18]. Two thirds of the patients in the open group were ASA-classification three patients compared to just over one third in the laparoscopic group. The significant age difference between groups may have influenced outcomes and reflects potential selection bias.

In the past, several studies have shown that, laparoscopic procedures in the emergency setting are more often performed in centres with dedicated colorectal emergency services [17, 19–21]. Although 42.5% of patients were operated on during on-call hours, the majority (71.2%) were performed by specialized colorectal surgeons.

It could be hypothesized that operative times would be longer when performing laparoscopy in patients with obstructed colorectal disease. However, contrasting numbers have been published on this matter, making it hard to evaluate the validity of this finding [11]. In the present study, operative times did not differ between the laparoscopic and the open surgery group.

A negative resection margin (R0) is an established prognostic factor for local recurrence and disease-free survival in colorectal cancer surgery. R0 rates did not significantly differ between the laparoscopic and the open surgery group. The LN yield in patients with malignant obstruction did differ between the groups, however, although lymph-node yield was higher in the laparoscopic group, this finding should be interpreted with caution. Lymph-node yield may be influenced by several factors, including pathology practices, tumour location, extent of resection, centre-specific factors, and surgeon subspecialisation. Given the observational design and limited sample size, the observed difference cannot be interpreted as evidence of oncological superiority of the laparoscopic approach. The present study focused on short-term perioperative outcomes and did not include sufficient follow-up to evaluate long-term oncological outcomes such as disease-free survival, overall survival, or local recurrence. In addition, the limited sample size precluded meaningful subgroup analyses according to obstruction aetiology and anatomical location.

### Limitations

Strengths of this study include the multicentre design and inclusion of contemporary patients from two healthcare systems. However, as patients were recruited from three centres in two healthcare systems, centre-level differences in patient selection, perioperative management, discharge practices, pathology assessment, and surgeon availability may have influenced outcomes. Owing to the limited sample size, adjustment for centre effects was not considered statistically robust and residual confounding from centre-specific factors cannot be excluded. Furthermore, the unequal distribution of laparoscopic surgery across participating centres further suggests that institutional practices and surgeon availability may have influenced treatment allocation.

The retrospective design of the present study is its principal limitation. Retrospective observational studies are inherently subject to selection bias, as the surgical approach was determined by the surgeon on call, potentially reflecting patient condition, surgeon preference, or institutional logistics. Furthermore, the relatively small sample size limited the number of covariates that could be included in multivariable analyses. Additionally, complete separation between laparoscopic surgery and colorectal surgeon involvement precluded meaningful propensity score matching. As a result, residual confounding by measured and unmeasured factors remains likely despite multivariable adjustment. Similarly, the LOS analyses should be considered exploratory. Alternative statistical approaches, including competing-risk or accelerated failure-time models, may have yielded different estimates; however, the limited sample size restricted the feasibility and robustness of more complex modelling strategies.

Reliance on existing medical records also entails risk of information bias due to incomplete or inconsistent documentation, while missing data may confound comparisons between groups. As postoperative complications were collected retrospectively from the index admission, complications diagnosed after discharge may have been underreported. Additionally, the limited number of events precluded meaningful analysis of specific complication subtypes. Moreover, heterogeneity in perioperative management and lack of standardized follow-up could have influenced documentation of outcomes. Despite our countermeasures such as rigorous data validation and predefined inclusion criteria, the study design precludes us from drawing anything but cautious conclusions.

## Conclusions

In this selected multicentre cohort, laparoscopic emergency colonic resection was associated with reduced complication rates, shorter LOS, and less intensive postoperative analgesic requirements. However, these findings should be interpreted cautiously and considered exploratory given substantial differences in baseline patient characteristics and surgeon specialization between groups. The observed associations may reflect patient selection, surgeon expertise, and other unmeasured confounders. The results merit validation in well-designed prospective and appropriately powered studies.

## Supplementary Information

Supplementary Material 1.

Supplementary Material 2.

## Data Availability

The datasets generated and/or analysed during the current study are not publicly available due to institutional and ethical restrictions but are available from the corresponding author on reasonable request and with permission from the relevant authorities.

## Abbreviations

ASA: American Society of Anaesthesiologists
BMI: Body Mass Index
CCI: Comprehensive Complication Index
CD: Clavien–Dindo
IQR: Interquartile Range
LN: Lymph Node
LOS: Length of Stay
PCA: Patient-Controlled Analgesia
PCEA: Patient-Controlled Epidural Analgesia

## References

[1] Govaert JA, Fiocco M, van Dijk WA, Kolfschoten NE, Prins HA, Dekker J-WT, et al. Multicenter Stratified Comparison of Hospital Costs Between Laparoscopic and Open Colorectal Cancer Resections: Influence of Tumor Location and Operative Risk. Ann Surg. 2017;266:1021–8. 10.1097/SLA.0000000000002000. 27611610

[2] Schwenk W, Haase O, Neudecker J, Müller JM. Short term benefits for laparoscopic colorectal resection. Cochrane Database Syst Rev. 2005;2005:CD003145. 10.1002/14651858.CD003145.pub2. 16034888 PMC8693724

[3] Lacy AM, García-Valdecasas JC, Delgado S, Castells A, Taurá P, Piqué JM, et al. Laparoscopy-assisted colectomy versus open colectomy for treatment of non-metastatic colon cancer: a randomised trial. Lancet. 2002;359:2224–9. 10.1016/S0140-6736(02)09290-5. 12103285

[4] Abraha I, Binda GA, Montedori A, Arezzo A, Cirocchi R. Laparoscopic versus open resection for sigmoid diverticulitis. Cochrane Database Syst Rev. 2017;11:CD009277. 10.1002/14651858.CD009277.pub2. 29178125 PMC6486209

[5] Sica GS, Iaculli E, Benavoli D, Biancone L, Calabrese E, Onali S, et al. Laparoscopic versus open ileo-colonic resection in Crohn’s disease: short- and long-term results from a prospective longitudinal study. J Gastrointest Surg. 2008;12:1094–102. 10.1007/s11605-007-0394-6. 18027061

[6] Clinical Outcomes of Surgical Therapy Study Group, Nelson H, Sargent DJ, Wieand HS, Fleshman J, Anvari M, et al. A comparison of laparoscopically assisted and open colectomy for colon cancer. N Engl J Med. 2004;350:2050–9. 10.1056/NEJMoa032651. 15141043

[7] Ingraham AM, Cohen ME, Bilimoria KY, Feinglass JM, Richards KE, Hall BL, et al. Comparison of hospital performance in nonemergency versus emergency colorectal operations at 142 hospitals. J Am Coll Surg. 2010;210:155–65. 10.1016/j.jamcollsurg.2009.10.016. 20113935

[8] Bakker IS, Snijders HS, Grossmann I, Karsten TM, Havenga K, Wiggers T. High mortality rates after nonelective colon cancer resection: results of a national audit. Colorectal Dis. 2016;18:612–21. 10.1111/codi.13262. 26749028

[9] Clavien PA, Barkun J, De Oliveira ML, Vauthey JN, Dindo D, Schulick RD, et al. The Clavien-Dindo classification of surgical complications: five-year experience. Ann Surg. 2009;250:187–96. 10.1097/SLA.0B013E3181B13CA2. 19638912

[10] Slankamenac K, Graf R, Barkun J, Puhan MA, Clavien P-A. The Comprehensive Complication Index. Ann Surg. 2013;258:1–7. 10.1097/SLA.0b013e318296c732. 23728278

[11] Moghadamyeghaneh Z, Talus H, Ballantyne G, Stamos MJ, Pigazzi A. Short-term outcomes of laparoscopic approach to colonic obstruction for colon cancer. Surg Endosc. 2021;35:2986–96. 10.1007/s00464-020-07743-w. 32572627

[12] Cirocchi R, Cesare Campanile F, Di Saverio S, Popivanov G, Carlini L, Pironi D, et al. Laparoscopic versus open colectomy for obstructing right colon cancer: A systematic review and meta-analysis. J Visc Surg. 2017;154:387–99. 10.1016/j.jviscsurg.2017.09.002. 29113714

[13] Warps A-LK, Zwanenburg ES, Dekker JWT, Tollenaar RAEM, Bemelman WA, Hompes R, et al. Laparoscopic Versus Open Colorectal Surgery in the Emergency Setting: A Systematic Review and Meta-analysis. Ann Surg Open. 2021;2:e097. 10.1097/AS9.0000000000000097. 37635817 PMC10455067

[14] Gietelink L, Wouters MWJM, Bemelman WA, Dekker JW, Tollenaar RAEM, Tanis PJ, et al. Reduced 30-Day Mortality After Laparoscopic Colorectal Cancer Surgery: A Population Based Study From the Dutch Surgical Colorectal Audit (DSCA). Ann Surg. 2016;264:135–40. 10.1097/SLA.0000000000001412. 27272958

[15] Ballian N, Weisensel N, Rajamanickam V, Foley EF, Heise CP, Harms BA, et al. Comparable postoperative morbidity and mortality after laparoscopic and open emergent restorative colectomy: outcomes from the ACS NSQIP. World J Surg. 2012;36:2488–96. 10.1007/s00268-012-1694-x. 22736343

[16] Lee YF, Brown RF, Battaglia M, Cleary RK. Laparoscopic Versus Open Emergent Sigmoid Resection for Perforated Diverticulitis. J Gastrointest Surg. 2020;24:1173–82. 10.1007/s11605-019-04490-9. 31845141

[17] Vallance AE, Keller DS, Hill J, Braun M, Kuryba A, van der Meulen J, et al. Role of Emergency Laparoscopic Colectomy for Colorectal Cancer: A Population-based Study in England. Ann Surg. 2019;270:172–9. 10.1097/SLA.0000000000002752. 29621034

[18] Sermonesi G, Tian BWCA, Vallicelli C, Abu-Zidan FM, Damaskos D, Kelly MD, et al. Cesena guidelines: WSES consensus statement on laparoscopic-first approach to general surgery emergencies and abdominal trauma. World J Emerg Surg. 2023;18:57. 10.1186/s13017-023-00520-9. 38066631 PMC10704840

[19] Osagiede O, Haehn DA, Spaulding AC, Otto N, Cochuyt JJ, Lemini R, et al. Influence of surgeon specialty and volume on the utilization of minimally invasive surgery and outcomes for colorectal cancer: a retrospective review. Surg Endosc. 2021;35:5480–8. 10.1007/s00464-020-08039-9. 32989545

[20] Archampong D, Borowski D, Wille-Jørgensen P, Iversen LH. Workload and surgeon’s specialty for outcome after colorectal cancer surgery. Cochrane Database Syst Rev. 2012;2012:CD005391. 10.1002/14651858.CD005391.pub3. 22419309 PMC12076000

[21] Keller DS, Pedraza R, Flores-Gonzalez JR, LeFave JP, Mahmood A, Haas EM. The current status of emergent laparoscopic colectomy: a population-based study of clinical and financial outcomes. Surg Endosc. 2016;30:3321–6. 10.1007/s00464-015-4605-z. 26490770

