# Supplementary material for: Laparoscopic versus open emergency colonic resection for obstruction: a multicentre retrospective cohort study
Source: BMC Surg. 2026 Sep 18;26:622. doi: 10.1186/s12893-026-04220-4 (PMC13587340; doi:10.1186/s12893-026-04220-4)
Supplement: Supplementary file 1 [file 12893_2026_4220_MOESM1_ESM.docx]

Supplement 1 - STROBE Checklist

| Item | Recommendation | Reported on page |
| --- | --- | --- |
| 1(a) | Indicate study design in title or abstract | Title; Abstract |
| 1(b) | Provide informative summary | Abstract |
| 2 | Scientific background and rationale | Introduction |
| 3 | Objectives and hypotheses | Introduction |
| 4 | Key elements of study design | Methods |
| 5 | Setting, locations, dates | Methods |
| 6(a) | Eligibility criteria and participant selection | Methods |
| 7 | Define outcomes/exposures/confounders | Methods |
| 8 | Data sources and assessment | Methods |
| 9 | Address sources of bias | Methods; Discussion |
| 10 | Explain study size | Statistical analysis |
| 11 | Handling of quantitative variables | Statistical analysis |
| 12(a) | Statistical methods and confounding control | Statistical analysis |
| 12(c) | Missing data handling | Data collection |
| 12(e) | Sensitivity analyses | Results |
| 13(a) | Numbers at each stage | Results; Figure 1 |
| 13(b) | Reasons for exclusion | Figure 1 |
| 14(a) | Participant characteristics | Table 1 |
| 14(b) | Missing data by variable | Tables 1–2 |
| 15 | Outcome events reported | Tables 2–3 |
| 16(a) | Unadjusted and adjusted estimates | Results; Supplementary Tables |
| 17 | Other analyses/sensitivity analyses | Results |
| 18 | Summary of key results | Discussion |
| 19 | Limitations | Discussion |
| 20 | Interpretation of results | Discussion; Conclusion |
| 21 | Generalizability | Discussion |
| 22 | Funding | Funding statement |
