# Supplementary material for: Laparoscopic versus open emergency colonic resection for obstruction: a multicentre retrospective cohort study
Source: BMC Surg. 2026 Sep 18;26:622. doi: 10.1186/s12893-026-04220-4 (PMC13587340; doi:10.1186/s12893-026-04220-4)
Supplement: Supplementary file 2 [file 12893_2026_4220_MOESM2_ESM.docx]

| Cox proportional hazards regression - Length of stay | | | |
| --- | --- | --- | --- |
| Univariable | | | |
|  | *n* | HR (CI 95%) | p-value |
| Open resection | 39 | 1.00 | ref. |
| Laparoscopic resection | 30 | 1.76 (1.36-2.28) | <0.001 |
| Multivariable* | | | |
|  | *n* | HR (CI 95%) | p-value |
| Open resection | 39 | 1.00 | ref. |
| Laparoscopic resection | 30 | 1.37 (1.02-1.86) | 0.039 |
| Age | 30 | 0.97 (0.54-0.98) | 0.008 |
| Sex | 30 | 0.97 (0.74-1.28) | 0.844 |
| ASA 1 | 30 | 8.61 (1.39-53.55) | 0.021 |
| ASA 2 | 30 | 3.37 (0.64-17.84) | 0.152 |
| ASA 3 | 30 | 2.30 (0.47-11.30) | 0.304 |
| ASA 4 | 30 | 1.79 (0.24-13.14) | 0.566 |

Supplement 2. HR, hazard ratio; OR, odds ratio; CI, confidence interval; ASA, American Society of Anaesthesiologists. HR >1 indicates a higher probability of discharge, whereas OR >1 indicates higher odds of postoperative complications. Multivariable models were adjusted for age, sex, and ASA classification.
